# Microplate-reader method for the rapid analysis of copper in natural waters with chemiluminescence detection

**DOI:** 10.3389/fmicb.2012.00437

**Published:** 2013-01-17

**Authors:** Axel Durand, Zanna Chase, Tomas Remenyi, Fabien Quéroué

**Affiliations:** ^1^Institute for Marine and Antarctic Studies, University of TasmaniaHobart, TAS, Australia; ^2^Antarctic Climate and Ecosystems Cooperative Research Centre, University of TasmaniaHobart, TAS, Australia; ^3^Australian Centre for Research on Separation Science, School of Chemistry, University of TasmaniaHobart, TAS, Australia; ^4^Université Européenne de BretagneRennes, France; ^5^Université de Brest, CNRS, IRD, UMR 6539/LEMAR/IUEM, Technopôle Brest IroisePlouzané, France

**Keywords:** microplate-reader, copper detection, chemiluminescence, 1,10-phenanthroline, seawater

## Abstract

We have developed a method for the determination of copper in natural waters at nanomolar levels. The use of a microplate-reader minimizes sample processing time (~25 s per sample), reagent consumption (~120 μL per sample), and sample volume (~700 μL). Copper is detected by chemiluminescence. This technique is based on the formation of a complex between copper and 1,10-phenanthroline and the subsequent emission of light during the oxidation of the complex by hydrogen peroxide. Samples are acidified to pH 1.7 and then introduced directly into a 24-well plate. Reagents are added during data acquisition via two reagent injectors. When trace metal clean protocols are employed, the reproducibility is generally less than 7% on blanks and the detection limit is 0.7 nM for seawater and 0.4 nM for freshwater. More than 100 samples per hour can be analyzed with this technique, which is simple, robust, and amenable to at-sea analysis. Seawater samples from Storm Bay in Tasmania illustrate the utility of the method for environmental science. Indeed other trace metals for which optical detection methods exist (e.g., chemiluminescence, fluorescence, and absorbance) could be adapted to the microplate-reader.

## Introduction

Copper, like many trace metals, is an essential micronutrient at very low concentrations or availability, but may be toxic and have deleterious effects at elevated concentrations. Copper concentrations in natural waters vary greatly depending on water type and location. Open ocean surface water concentrations can be as low as 0.5 nM, while deep ocean concentrations are as high as 5 nM (Boyle et al., 1977; Bruland, 1980). Coastal waters generally have higher copper concentrations, from between 1 nM in pristine waters to as high as 755 nM in highly contaminated coastal area such as Chañaral in northern Chile (Stauber et al., 2005) or 170 nM for the Erhjin Chi Esturay in China (Han et al., 1994). The average concentration of copper in world rivers is 160 nM (Sarmiento and Gruber, 2006), with concentrations as high as 1.2 mM found in the Odiel river, Spain (Olias et al., 2004).

Overexposure to high copper concentrations is associated with myriad biological and ecological impacts, including salmon physiology (Baldwin et al., 2003), domoic acid production by toxigenic pennate diatoms (Maldonado et al., 2002), and the growth and relative abundance of phytoplankton species (Moffett et al., 1997; Mann et al., 2002; Paytan et al., 2009). Mining, fossil fuel combustion, industrial processes, and other anthropogenic activities have greatly accelerated the release of this toxic trace metal to the oceans (Newman and Unger, 2003) resulting in increased efforts to determine the sources, transport and fate of Cu (and other metals) in the aquatic environment (Taylor and Shiller, 1995).

Current methods for low-level copper analysis in natural waters include flow injection analysis with chemiluminescence detection (Coale et al., 1992) based on the luminescence produced by the complexation of copper with 1,10-phenanthroline (Yamada and Suzuki, 1984), *in-situ* analysis using chemiluminescence (Holm et al., 2008) and colorimetry (Callahan et al., 2003), ICP-MS (Field et al., 1999) and electrochemical methods (Achterberg and Braungardt, 1999; Wang, 2002). The complexity of these methods and their relatively large sample size requirements (generally at least 40 mL) has limited their use and contributes directly to the lack of regular monitoring of environmental copper concentrations. Such measurements would increase our understanding of the sources of copper to the aquatic environment and their impact. A simple, low-volume, and low-cost method for copper analysis would also be useful in manipulative biological experiments such as *in-vitro* culturing work (Brand et al., 1986; Peers et al., 2005) and mesocosm incubations (Paytan et al., 2009) where sample volumes are often limited.

Microplate-readers, or plate-readers, are instruments designed to measure the absorbance, fluorescence, or luminescence of samples in microtitre plates. The plates typically consist of 96 wells, with a volume of 100–200 μL per well. Their main advantages are small sample volume, high-throughput of samples and ease of use. They have been widely used in biological sciences for many years (Ashour et al., 1987), but their use in environmental chemistry is limited. Methods have been published for macro-nutrient analysis in seawater (Ringuet et al., 2011) and pore-water constituents (Laskov et al., 2007). To our knowledge there have been no plate-reader methods developed for low-level trace metal analysis.

In this paper we describe the development of a plate-reader method to detect copper by chemiluminescence via the reaction with 1,10-phenanthroline and hydrogen peroxide. The plate reader is well suited to this application. Many chemiluminescent reactions are kinetically fast, resulting in optimal detection immediately after reagent-sample mixing. The plate reader uses two precision injectors (two needles placed around the detector) to introduce reagent into the sample and is able to begin detection before or during reagent injection. Furthermore, the small size of the instrument makes it practical for use at sea or in the field.

## Materials and methods

### Materials

This work used a FLUOstar OPTIMA microplate-reader equipped with two reagent injectors. The reagent needles are made of stainless steel, the tubing and valve housing are made of Teflon and Kel-F, and the syringe barrel is made of glass. Each syringe has a volume of 500 μL, and can inject a minimum of 50 μL and maximum of 450 μL in each cell. The detector present on the FLUOstar OPTIMA is a photomultiplier tube (PMT). No filters are used in this method.

All microplate measurements were made in a lab with an air conditioning system set to 20°C. No further temperature control was employed.

Low Density Polyethylene (LDPE) bottles were used to store reagents, stock solutions and samples. Plates used to determine copper concentration were the CELLSTAR Cell Culture Multiwell Plates 24 and (grenier bio-one) with a physical surface treatment, made in crystal Clear® polystyrene, sterile and individually packed.

### Measures to minimize contamination

The preparation and manipulation of the reagents, standards, and samples was carried out in a class 100 laminar flow hood. All the materials used for the experiments (e.g., reagents, samples, standards, tips) were stored in two ziplock bags inside the flow hood except for the hydrogen peroxide, which was stored in three ziplock bags in a fridge dedicated to trace metal analysis. Not ideally, the microplate reader instrument was not in a laminar flow hood, so plates and reagents were placed in a ziplock bag for transport and kept covered until analysis. During the analysis reagent bottles were partially capped and kept in closed ziplock bags. During manipulations the operator wore non-sterile nitrile gloves.

New and used bottles were rinsed five times with Milli-Q water, and then soaked in a 6 N HCl solution (Aldrich ACS reagent). Then the bottles were rinsed five times with Milli-Q water and soaked for 7 days in a 6 N trace metal grade nitric acid (J. T. Baker) solution. Then bottles were rinsed 10 times with Milli-Q water, dried in a laminar flow hood, and stored capped in ziplock bags. Acid baths were changed after 6 months or 200 bottles washed. Pipet tips were cleaned immediately prior to use with two rinses of 6 N Ultrex® II HCl, one rinse of Milli-Q water then 1–3 complete volumes of the intended solution.

Plates were rinsed first with 50% acetone (Aldrich ACS reagent), and then rinsed five times with Milli-Q water. The cells were then filled with 6 N trace metal HCl (J. T. Baker) for two days. Finally they were rinsed five times with Milli-Q water and dried in a laminar flow hood. Plates were used only once because during prolonged exposure the hydrogen peroxide attacked the plastic.

The two precision injectors of the plate reader, used to inject reagents, were washed each day before use. The first step was to rinse the syringes, needles, and tubes three times with 4.5 mL (this is the maximum volume of the syringe) of 6 N HCl (J. T. Baker “trace metal” grade). Then injectors were rinsed three times with 4.5 mL of Milli-Q. These steps were repeated and finally the injectors were flushed with 4.5 mL of each reagent. At the end of each analytical session the system was rinsed two times with 4.5 mL of Milli-Q water.

### Analytical method

A chemiluminescence method for flow-through analysis of copper (II) in seawater using 1,10-phenanthroline (Coale et al., 1992; Zamzow et al., 1998), was adapted to the microplate-reader. This method involves the production of luminescence during the catalytic decomposition of hydrogen peroxide by the copper-1,10-phenanthroline complex, at a pH ~9.5 (Yamada and Suzuki, 1984). The addition of cetylethyldiethylammonium bromide (CEDAB) introduces surfactant micelles in solution (Yamada and Suzuki, 1984) which increases the sensitivity of the method by increasing the probability of contact between the reagent and the dissolved copper in the solution. In addition, a stable complexing agent for copper, tetraethylenepentamine (TEPA), is added to remove the background signal attributed to copper impurities in the reagents themselves (Yamada and Suzuki, 1984).

### Reagent preparation

Reagent 1 consists of 30% (in volume) hydrogen peroxide solution (Stabilized ACS reagent grade).

Reagent 2 contains 0.180 M 1,10-phenanthroline, 0.06 M TEPA, 0.225 M NaOH and 0.06 M CDAB in purified Milli-Q water (see below). This reagent is made by adding 2.4 g of CDAB (Reagent grade, Sigma) and 0.9 g of NaOH (ACS Reagent grade, J. T. Baker) to 100 mL of purified Milli-Q water and allowing for complete dissolution. Then 30 μL of a 4 mM stock solution of TEPA (97%, Fluka) and 1.5 mL of a 12 mM stock solution of 1,10-phenanthroline (99%, Aldrich) are added.

### Creation of standards and blanks

All standard and blank solutions are made from “copper free” seawater or Milli-Q water. Seawater is filtered using a 0.2 μm poly-ester-sulphone membrane filter (Pall Acropak). The filtered water is brought to pH 6 with ~300 μL/L of 6 N Ultrex® II HCl and passed through a 5 mL iminodiacetic acid column (HiTrap Chelating HP, Amersham Biosciences) at a flow rate of <5 mL/min. The column is prepared by first washing it with 50 mL of 0.1 N HCl, then 100 mL of Milli-Q water, and finally with 50 mL of seawater before collection. Acidified (pH ~1.7) standards are made from pH 6 seawater which is then acidified by adding 3.7 mL/L of 6 N Ultrex® II HCl. Standards are prepared by gravimetric dilution from a 1 g/L copper certified reference solution for trace metal analysis and were stable for at least 1 month. Milli-Q water cleaned with the HiTrap column (“purified Milli-Q”), was used to make pure water standards and blanks.

### Procedure

The cleaned and dried 24-well plate is filled with 700 μL of pH 1.7 sample, blanks and standards, using a 100–1000 μL micropipette and clear polypropylene tips. Generally five pH 1.7 standards and a blank, bracketing the range of expected concentrations, are used, with four replicates of each standard. We generally run each sample in triplicate. The plate is covered and placed inside two ziplock bags for transport to the plate reader. The plate reader injectors are flushed (see cleaning process) and finally the plate is loaded into the plate reader and analyzed. Generally samples are inside the plate for less then an hour before their analysis.

One hundred and twenty microliter of each reagent is injected to produce the luminescence reaction. A pump speed of 310 μL/sec was used, as recommended by the manufacturer. This speed permits good mixing between the reagents and the sample and limits well-to-well contamination. The positioning delay refers to a waiting period after a well of the microplate moves to the measurement position and before the measurement begins. The positioning delay allows the liquid to settle and the surface to become stable so that the measurement is more accurate. The recommended delay time from BMG LABTECH of 0.2 s was used. An end-point mode was used for all analyses, meaning each cell was measured once before moving to the next cell. The instrument was programmed to record light emission after 25 s, in order to maximize the signal (see “Results and Discussion”).

### Sample collection

Surface seawater was collected from six stations in Storm Bay, Tasmania, from a 15 m aluminum ship. A nylon net was braided around a 60 mL acid-washed LDPE bottle to create a holder and the net was linked to the operator with a 5 m nylon line. Eight 30 g lead weights placed inside ziplock bags were attached to the net to sink the bottle. To keep the bottle as far as possible from the boat hull a boat hook was used to catch the line, separated from the sample bottle by at least a meter in order to not contaminate the sample. Contact between sample and air never exceed 30 s (the time to catch and close the bottle). All samples were individually packed inside two ziplock bags and stored in a closed HDPE box for transport back to the lab. The nylon net was rinsed with 1 M HCl and Milli-Q water between samples. Samples were refrigerated for two days, then acidified to pH 1.7 with ultra-pure HCl and then filtered through acid-cleaned 0.2 μm poly-ester-sulphone membrane filters (pall Acropak). This sequence (acidification followed by filtration) is not ideal, and consequently the copper concentrations reported here should be interpreted with caution. They most likely represent a fraction equivalent to slightly less than the total dissolvable copper.

### UV irradiation

We investigated the impact of UV irradiation on copper analysis. Samples were dispensed into 100 mL teflon bottles and placed between two commercial GPH843TSL/4 ultraviolet lamps inside a black PVC chamber for 1–3 h.

### Calculation

The detection limit is calculated as three times the standard deviation of the concentration measured on blanks. Precision is calculated as the relative standard deviation (%) (RSD) of the standard concentration. Generally analyses were performed on four replicates, except where noted.

## Results and discussion

### Repeatability

**Figure 1:**
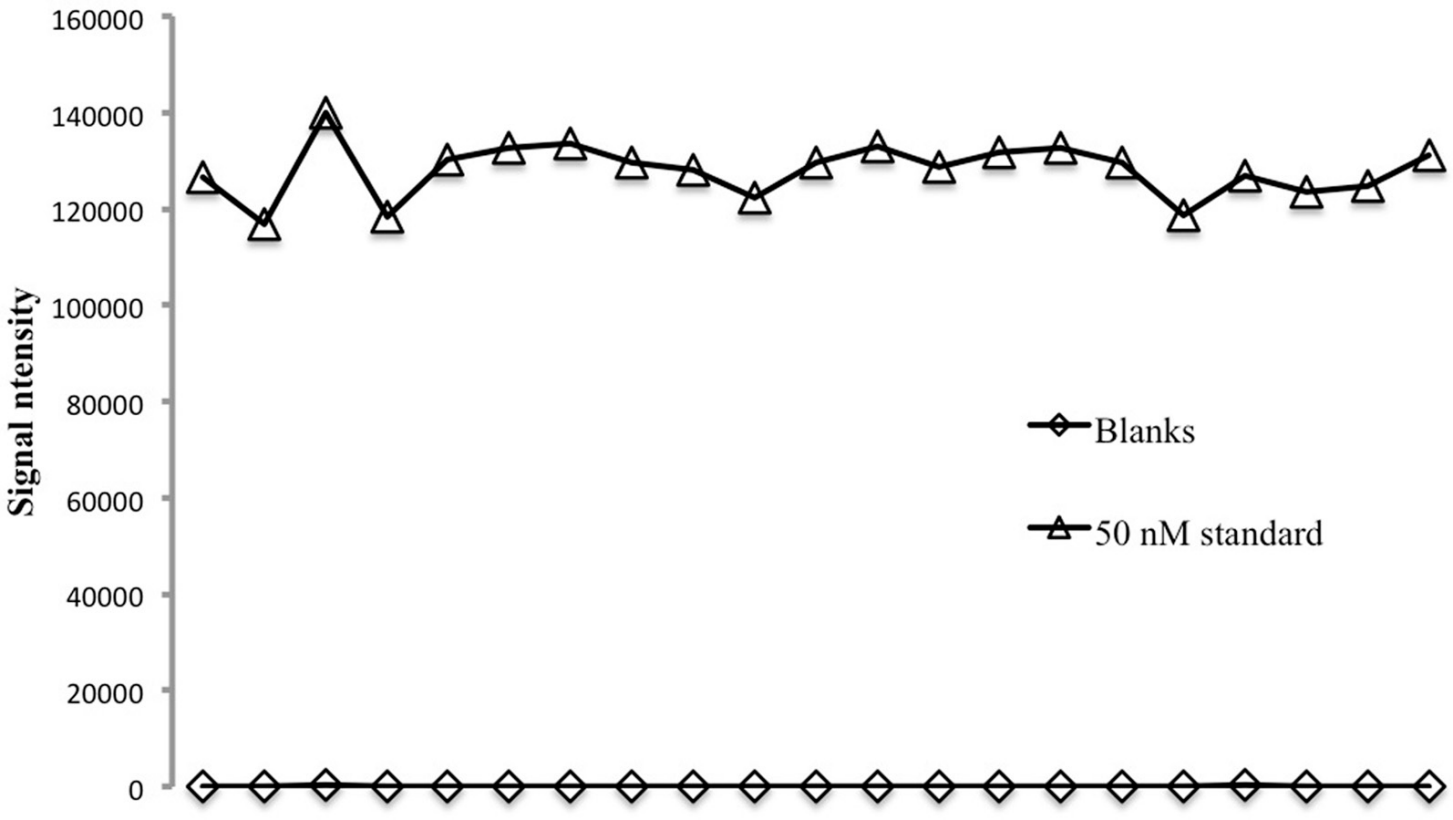
**Reproducibility on 21 replicates analyzed in a 24-well plate for 50 nM seawater consistency standard and seawater blanks**.

The repeatability of the method was assessed using a 24-well plate filled with 700 μL of either a 50 nM seawater standard or copper-free seawater. The Cu-free seawater was obtained by passing a low-Cu, UV-irradiated open ocean surface sample through the chelating column. For each plate the first three replicates were discarded because of poor precision on start-up. Well-to-well reproducibility is good (Figure 1), with a relative standard deviation of 4.46% on the 50 nM standard or a standard deviation equivalent to 2.23 nM. On blanks the standard deviation is equivalent to 0.23 nM. Generally the %RSD of replicates run on the same plate is about 5% and never above 10%.

### Sensitivity, linear range, detection limits, and accuracy

**Figure 2:**
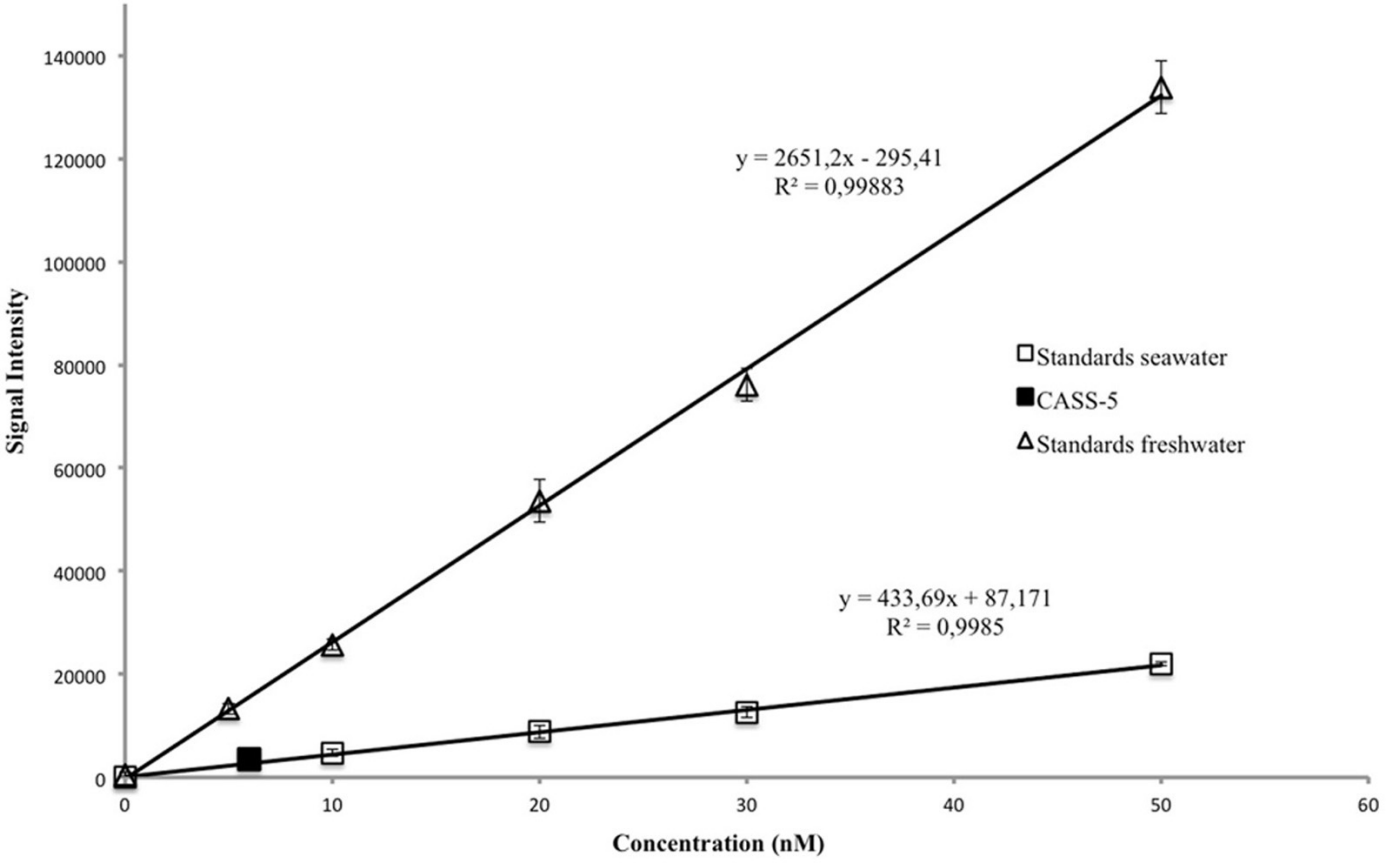
**Calibration curves in seawater and freshwater with CASS-5 reference for seawater**.

Detection limits were 0.4 nM in pure water and 0.7 nM in seawater (Figure 2). Linearity was observed to 200 nM in pure water and 100 nM in seawater (data not shown). Sensitivity, represented by the slope of the calibration curve, was always greater in pure water then in seawater by a factor of 3–5, consistent with previous studies (Holm et al., 2008). It is therefore important to match the standard matrix to that of the samples. Sensitivity varied by 25% day-to-day and appeared random, possibly linked to factors such as room temperature, humidity, oxygen, and CO_2_ levels (Xiao et al., 2002). It is therefore important to perform a calibration with each plate analysed.

Accuracy was assessed with four replicates of the standard reference material CASS-5 (5.9 ± 0.44 nM). The measured value for CASS-5 (7.1 ± 1.1 nM) is consistent with the certified value (Figure 2), confirming the accuracy of the technique.

### Optimization of injected reagent concentrations

**Figure 3:**
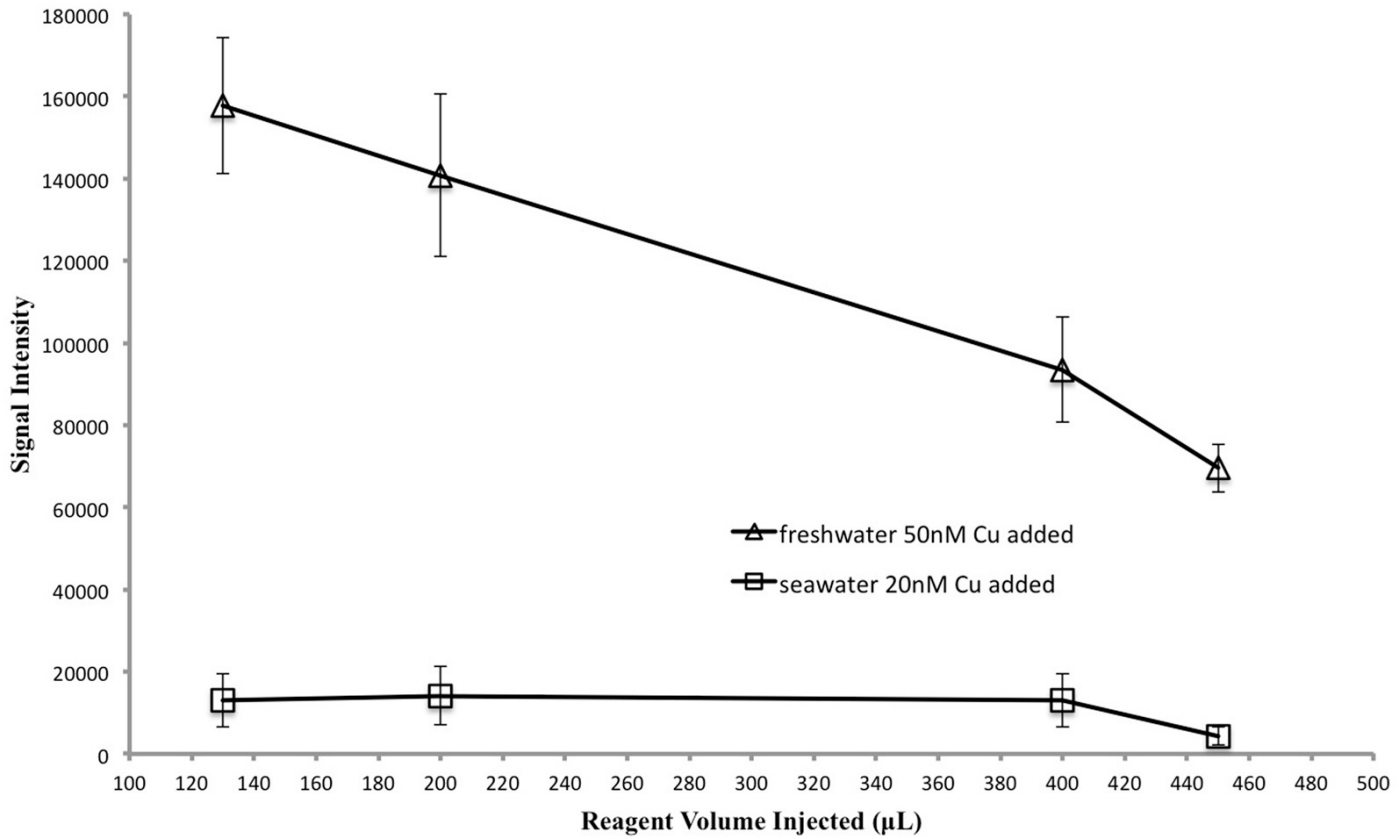
**Reagent volume vs. signal intensity to test the optimum reagent concentration and volume.** Sample volume was held constant. As reagent volume was adjusted so too were reagent concentrations, in order to maintain a constant amount of each reagent in the cell. See Table 1 for details.

Previous studies (Zamzow et al., 1998; Holm et al., 2008) have optimized reaction pH, and concentrations of 1,10-phenanthroline, TEPA, CDAB, and hydrogen peroxide for maximum signal intensity. We wanted to see whether the concentration of reagents injected into the cells had an impact on signal intensity. We were interested in minimizing contamination and reagent volume injected. In this test we maintained optimized reagent concentrations and pH in the reaction cells but adjusted the volume and concentration of injected reagents; when the reagent concentrations were increased the volume injected was decreased proportionally within the capabilities of the plate reader's injectors (Table 1). Sample volume was held constant at 700 μL. In pure water, the signal intensity increased with decreasing reagent volume, reaching a maximum at 130 μL (Figure 3), corresponding to a hydrogen peroxide concentration of 30%. This result most likely reflects the effect of a decreased dilution of the sample. In seawater the signal intensity was not as sensitive to reagent volume (Figure 3). However, using a 30% hydrogen peroxide solution and 130 μL reagent injection for both pure water and seawater reduces the risk of contamination by eliminating a dilution step during reagent preparation.

**Table 1:** **Reagent and sample volumes used in the experiment to optimize reagent concentrations and volumes**.

**Reagent volume injected (μL)**	**Sample volume (μL)**	**R1 concentration in injectors (% V/V)**	**R1 amount of substance in cell (mmol)**	**R2 concentration in injectors (M)**	**R2 amount of substance in cell (μmol)**
130	700	30	1.3	0.18	24
200	700	20	1.3	0.12	24
400	700	10	1.3	0.06	24
450	700	5	0.75	0.03	13

The amount of substance of each reagent stays constant in the well by adjusting the volume and concentration injected.

### Optimization of reagent volume injected

**Figure 4:**
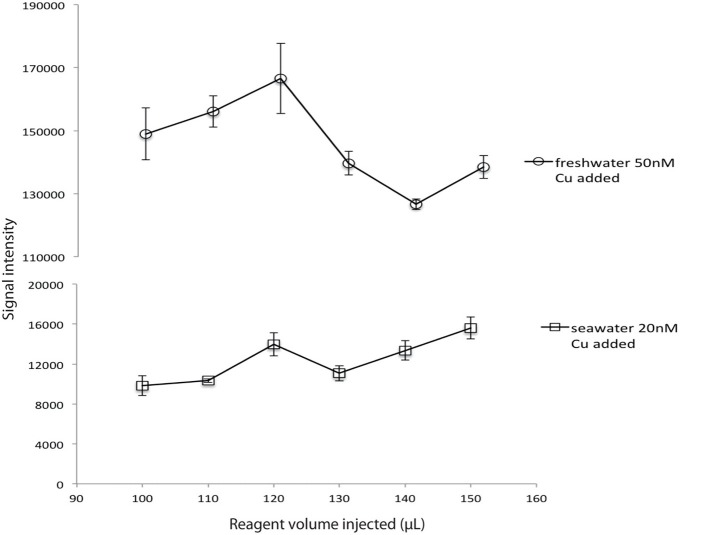
**Volume of reagents injected vs. signal intensity for a constant sample volume and a constant reagent concentration (R1 30% H_2_O_2_; R2 0.18 M)**.

An optimization was made to determine whether the assay response was sensitive to small variations in reagent volume. For this experiment reagent concentrations corresponding to 30% hydrogen peroxide were used (see Table 1) and the concentration of reagents in the well was not maintained constant; when the volume injected increased the concentration was not adjusted. For both seawater and freshwater, an injected volume of 120 μL produced the best signal intensity (Figure 4), so this volume was chosen for all experiments.

We also tried pre-mixing the two reagents in 1-1 proportions and using only one mixed reagent with twice the volume injected, as other studies have done. A mixed reagent did not work with the plate reader, as the two tensioactives (CDAB and TEPA) present in the phenanthroline reagent produced bubbles and foam inside the injector syringes in the presence of hydrogen peroxide.

### Optimization of detection time

**Figure 5:**
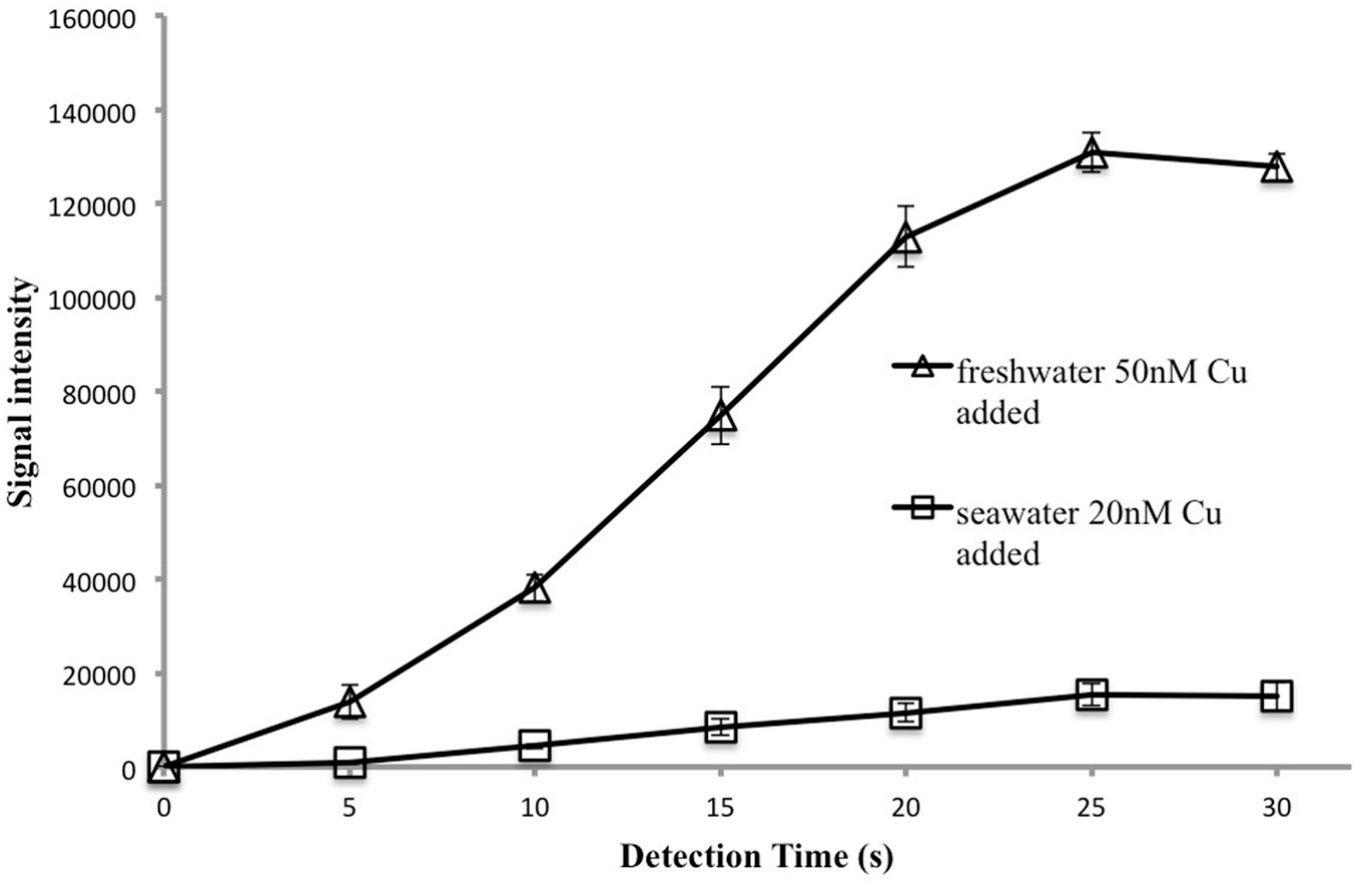
**Signal intensity as a function of detection time.** Each data point represents the average and standard deviation of 4 replicates analyzed after the time indicated. Thus four samples were analyzed after 5 s, another four after 10 s and so on.

The kinetics of the luminescence reaction between copper and 1,10-phenanthroline are very fast, less then a second (Eigen, 1963). However, we observed that signal intensity increased for 25 s after reagent injection (Figure 5). This delay probably occurs because there is no mechanical mixing in the well, so it takes time for convection and diffusion to achieve a homogenous solution and for complete reaction to occur. The optimal detection time was 25 s. For each cell, the luminescence signal at 25 s was recorded as the final signal. With these settings a 24-well plate can be analysed in 10 min.

### Plate contamination

We found that when new, sterile plates are used unwashed the signal intensity for blanks is as much as 35 nM and the standard deviation is more than 86% of the signal intensity. After 12 h in a cleaned plate the signal intensity of a 20 nM seawater standard (pH ~1.7) had increased by 45% and the standard deviation increased more than 4-fold. These data suggest that polystyrene continues to release copper into solution even if plates are acid-cleaned, consistent with previous studies (Batley and Gardner, 1977; Howard and Statham, 1997). Consequently plates need to be analysed as soon as possible after filling. The use of alternative plate materials such as PTFE or polyethylene may minimize this source of contamination.

### UV irradiation

UV irradiation is normally not used with flow-through copper detection via the luminescence reaction with 1,10-phenanthroline (Coale et al., 1992; Holm et al., 2008). An acidification step to pH ~1.7 for at least 24 h prior to analysis was assumed to release all ligand-bound copper to the solution. However, other studies have suggested UV irradiation may be necessary to dissociate some metal-ligand complexes (Van Den Berg, 1984; Achterberg et al., 2001a).

**Figure 6:**
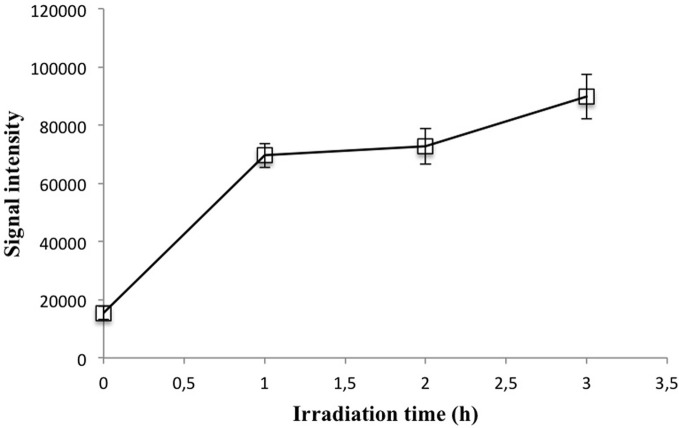
**Signal intensity vs. irradiation time for a 20 nM coastal seawater standard.** Each point represents the mean and standard deviation of four subsamples independently irradiated and analyzed.

We found evidence to support the need for UV irradiation with a seawater sample collected off the coast of Bruny Island, Tasmania. This water was sampled without respecting trace metal protocols, filtered, “copper-cleaned” with the Hi-Trap column, and acidified, after which copper was added to produce a range of standards. However, we were unable to obtain a linear calibration curve, with the number of photons emitted staying approximately constant across copper additions. Furthermore, the signal intensity for the un-amended sample was very high. It is clear that in this case the chelating column was not effective at removing copper ions, possibly due to presence of a strong ligand, as only free copper is removed by the column. Figure 6 shows the effect of UV irradiation on the signal intensity of a 20 nM addition made to this water. After 1 h of irradiation the signal intensity is increased 4-fold and stays approximately constant past this time, suggesting successful release from organic copper-binding ligands.

### Analysis of coastal seawater samples

**Figure 7:**
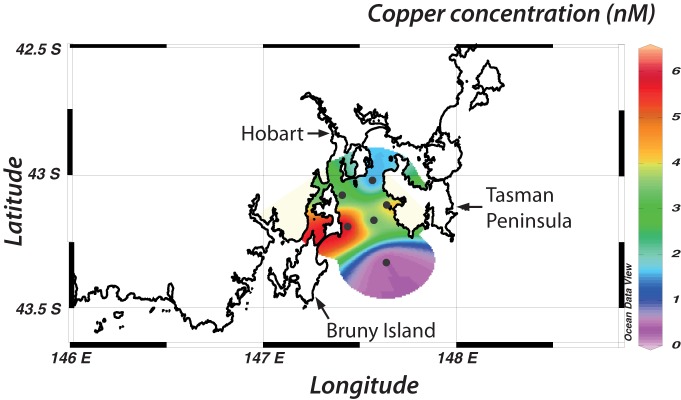
**Copper concentrations in Storm Bay, concentrations are given in nM.** Concentrations starting from the north east and moving south and east are 1.37 ± 0.4; 3.26 ± 0.7; 4.25 ± 0.8; 3.18 ± 1; 6.18 ± 0.4; 0.39 ± 0.1.

The analysis of six seawater samples collected from Storm Bay, Tasmania, illustrates the application of the plate-reader method to real samples (Figure 7). The sampling sites are directly downstream from the Derwent estuary, which is highly contaminated by trace metals including copper (Butler and Wangersky, 2006). Despite the proximity to the Derwent estuary, copper concentrations in surface waters of Storm Bay are relatively low, and similar to oceanic concentrations (0.3–3 nM) (Buckley and Van Den Berg, 1986; Coale and Bruland, 1990). One exception is a sample adjacent to Bruny Island with a concentration of 6.2 nM. We are not aware of any previous measurements of Cu in Storm Bay, but Butler and Wangersky (2006) report Cu concentrations in the Derwent River and estuary of 1.8–232 nM, consistent with our results. In the Macquarie estuary on the west coast of Tasmania, copper concentrations are between 10 and 50 nM (Carpenter et al., 1991). The relatively low copper concentrations measured in Storm Bay may be due to a strong oceanic influence, as a storm had passed through the area 3 days before sampling. This example demonstrates the efficiency of the plate reader method, as sample analysis took only 3 h, including acidification and filtration steps. The microplate-reader technique is particularly well suited to large sample sets (more than 100 samples); the absence of a pre-concentration step and the short detection time minimizes sample processing time.

### Potential applications

The plate-reader method for copper analysis described here provides a rapid, simple, low-volume method with good precision at relatively low concentrations suitable for work in freshwater and saltwater. These characteristics of the method make it applicable to a range of applied and basic research in the biological and environmental sciences. The method could be useful in culture, mesocosm and aquaculture studies and in the analysis of pore-waters or samples from benthic flux chambers. The method would also be useful for environmental monitoring of surface waters at high spatial and temporal resolution, or monitoring of wastewater streams and storm water overflow. Significantly, the detection limits of the method in freshwater and seawater are 13 and 7 times greater than the respective ANZECC guidelines for freshwater and marine ecosystems, 5.2 and 5.0 nM (ANZECC/ARMCANZ, 2000).

The detection limits reported here are not suitable for determining copper in seawater at the lowest concentrations found in the literature for the open ocean (e.g., <1 nM). This problem could be solved by the use of a dedicated, trace metal clean instrument placed inside a laminar flow hood to protect plates during their analysis and the use of other types of plates, for example polyethylene or teflon. The method could also be coupled with off-line pre-concentration (Biller and Bruland, 2012) for measurement of ultra-trace level samples.

Other optical methods for trace metal analysis using chemiluminescence, fluorescence, or spectrophotometric detection may be amenable to adaptation to the microplate reader. Iron (Achterberg et al., 2001b), cobalt (Sakamoto-Arnold and Johnson, 1987), aluminum (Hydes and Liss, 1976), and zinc (Grand et al., 2011), are good candidates for this method. A plate-reader method could also be used for Cu speciation, following the titration method of Zamzow et al. (1998).

## Conclusions

With the use of our optimization conditions, which include detection after 25 s, concentrated reagents with appropriate volume injected, and plate cleaning, we have obtained a low detection limit of 0.4 nM in freshwater and 0.7 nM in seawater for a 700 μL sample with a precision of 7%. These detection limits permit a rapid, simple, and inexpensive determination of copper concentration suitable for coastal seawater and river water.

### Conflict of interest statement

The authors declare that the research was conducted in the absence of any commercial or financial relationships that could be construed as a potential conflict of interest.
